# Effects of Phenotypic Variation on Biological Properties of Endophytic Bacteria *Bacillus mojavensis* PS17

**DOI:** 10.3390/biology11091305

**Published:** 2022-09-02

**Authors:** Roderic Gilles Claret Diabankana, Shamil Zavdatovich Validov, Alexandra Borisovna Vyshtakalyuk, Amina Daminova, Radik Ilyasovich Safin, Daniel Mawuena Afordoanyi

**Affiliations:** 1 Laboratory of Molecular Genetics and Microbiology Methods, Kazan Scientific Center of Russian Academy of Sciences, 420111 Kazan, Russia; 2 Centre of Agroecological Research, Kazan State Agrarian University, 420015 Kazan, Russia; 3 Laboratory of Structural Biology, Institute of Fundamental Medicine and Biology—Kazan (Volga Region) Federal University, 420021 Kazan, Russia; 4 Laboratory of Chemical-Biological Research, Arbuzov IOPC FRC Kazan Scientific Center, Russian Academy of Sciences, 420029 Kazan, Russia; 5 Kazan Institute of Biochemistry and Biophysics, Kazan Scientific Center of Russian Academy of Sciences, 420111 Kazan, Russia; 6 Tatar Scientific Research Institute of Agricultural Chemistry and Soil Science, FRC Kazan Scientific Center, Russian Academy of Sciences, 420111 Kazan, Russia

**Keywords:** endophyte bacteria, phenotypic variation, plant colonization, *B. mojavensis*, antagonistic activity, biological control

## Abstract

**Simple Summary:**

Microorganisms play an important role in agriculture by protecting and stimulating the growth of plants. The phenotypic activities of microbial biological agents (MBA) can change under different environmental conditions. However, to adapt to these harsh conditions, genetic mutations take place in bacteria that are seen phenotypically, which might not be beneficial or less beneficial to the plants. Some adaptative mechanisms used by microorganisms, especially bacteria, to face these environmental factors lead to the appearance of subpopulations with different morphotypes that may be more adapted to survive in stressful conditions. Moreover, in favorable conditions, these subpopulations may become dominant among the overall bacterial population. In this study, Bacillus mojavensis undergoes phase variation when grown in a minimal medium, in which two colonies, opaque (morphotype I) and translucent (morphotype II), were generated. The characteristics of the generated morphotypes were determined and compared with those of their original strain. Overall, the results obtained showed that the phenotypic characteristics of morphotype I statistically differed from morphotype II. This phenomenon may be one of the factors behind the dissimilarities in the results between the laboratory and field data on the application of MBA.

**Abstract:**

The use of microorganism-based products in agricultural practices is gaining more interest as an alternative to chemical methods due to their non-toxic bactericidal and fungicidal properties. Various factors influence the efficacy of the microorganisms used as biological control agents in infield conditions as compared to laboratory conditions due to ecological and physiological aspects. Abiotic factors have been shown to trigger phase variations in bacterial microorganisms as a mechanism for adapting to hostile environments. In this study, we investigated the stability of the morphotype and the effects of phenotypic variation on the biological properties of *Bacillus mojavensis* strain PS17. *B. mojavensis* PS17 generated two variants (opaque and translucent) that were given the names morphotype I and II, respectively. The partial sequence of the 16S rRNA gene revealed that both morphotypes belonged to *B. mojavensis*. BOX and ERIC fingerprinting PCR also showed the same DNA profiles in both morphotypes. The characteristics of morphotype I did not differ from the original strain, while morphotype II showed a lower hydrolytic enzyme activity, phytohormone production, and antagonistic ability against phytopathogenic fungi. Both morphotypes demonstrated endophytic ability in tomato plants. A low growth rate of the strain PS17(II) in a minimal medium was observed in comparison to the PS17(I) strain. Furthermore, the capacity for biocontrol of *B. mojavensis* PS17(II) was not effective in the suppression of root rot disease in the tomato plants caused by *Fusarium oxysporum* f. sp. *radices-lycopersici* stain ZUM2407, compared to *B. mojavensis* PS17(I), whose inhibition was almost 47.9 ± 1.03% effective.

## 1. Introduction

The heavy dependence on chemical antibiotics for crop protection in modern agriculture endangers the health of farm personnel and consumers of farm products. The harmful accumulation of these chemicals initiated the transition to the use of microbial agents for crop protection with most of them having low efficacy in infield experiments [1]. The unstable protective effects of biological control agents (BCA) can be explained by external factors, such as the influence of fluctuating environmental conditions and the resistance of phytopathogens to the biocontrol agent or endogenous microbiota, but also by the instability of the BCA. Phase variation, one of the mechanisms used by bacteria to adapt rapidly to changing environmental conditions, is a switching process mediated by mutations, reorganization, or DNA modification (methylation patterns, slipped-stand mispairings, transposition, duplication, deletion, and homologous recombination) resulting in reversible inclusion or change in surface phenotypes [2,3]. The occurrence of phenotypic variation is characterized by the appearance of different phenotypic subpopulations at a relative ratio normally exceeding 10^−5^ per growing cell per generation, as compared with spontaneous mutation, which occurs at a frequency of 10^−8^ to 10^−6^ [3,4]. Under appropriate conditions, these subpopulations can become the dominant type in the population [5]. This phenomenon has been reported in many bacteria belonging to different taxonomies, genera, and ecological behaviors [6,7]. Pathogenic bacteria such as *Haemophilus influenzae* and *Neisseria meningitidis* commonly use this mechanism to colonize their host, survive the immune response system, or develop antibiotic resistance [8,9].

Phenotypic variation has also been described in some microbial-based biocontrol uses in plant protection. For example, the phase variability of *Pseudomonas fluorescens* WCS365 caused a decrease in the strain’s ability to colonize the tomato root system [10,11], which was due to the loss of mobility. As an advantage of phase variability, some microorganisms use this phenomenon to create population diversity, which contributes to increased adaptation in ecological niches. In the work of Achouak et al. [12], it was observed that the colonization ability of the second phase variant of *Pseudomonas brassicacearum* NFM421 on the root system of *Arabidopsis thaliana* was better due to the ability to synthesize flagella. Li et al. [13] isolated an epiphytic strain of *Acidovorax radicis* from the roots of wheat that undergoes phase variability when grown in nutrient broth, with one variant losing its plant growth-promoting ability. Repeated inoculations and reverse isolation from maize roots of phase variability *Azospirillum brasilense* Sp7 obtained by constant growth under adverse conditions (starvation and stress) brought about variants [14]. These variants produced significantly more exopolysaccharides and were relatively different in composition as compared to the wild strain [14].

*Bacillus* species are frequently used as BCA due to their active secondary metabolites against several phytopathogens [15]. *B. mojavensis*, a closely related species to *Bacillus subtilis*, is an aerobic spore-forming and chemoorganoheterotrophic bacterium that is known for its biocontrol and plant growth-promoting activity [16]. One of the particular characteristics of the representatives of this species is an ability to produce ribosomal and nonribosomal cyclic peptide compounds and polyketides with diverse antifungal and antibacterial activities [17,18,19]. Numerous studies have reported the beneficial effects of this species on the plant growth of various crops, such as wheat, corn, soybeans, tomatoes, and others under laboratory conditions or in greenhouses [20,21].

Endophytic ability is a well-known characteristic of most *Bacillus* species and plays an important role in plant protection [22]. Endophytes protect plants by the secondary metabolites they produce in plant cells by direct interaction with the pathogens or by inducing the resistant system of the plant. To inhibit the growth of phytopathogenic bacteria, *Bacillus* spp. express quorum-quenching enzymes against quorum sensing (QS) or produce elicitors that activate plant defense systems mainly induced by systemic resistance (ISR) [23]. This property differs between species of the same genus [24] and also strains of the same species based on their habitat [25].

The *B. mojavensis* strain PS17 is a BCA-producing antagonistic compound that inhibits the growth of several phytopathogens [22]. We discovered phase variation in *B. mojavensis* PS17 and characterized their biological properties. In this study, we describe the phase variants of *B. mojavensis* PS17, compare the growth curve of the variants in a minimal and nutrient medium, and compare the production of exoenzyme and antifungal metabolite activity against phytopathogens. We also test their endophytic ability, and their capacity for biocontrol in tomato plants.

## 2. Materials and Methods

### 2.1. Bacterial and Fungal Strain Cultivation

The bacterial and fungal strains used in this work are given in Table 1. When needed, bacterial and fungal strains were plated from glycerol stock stored at −80 ± 1 °C. Czapec-Dox agar (CDA, Difco Laboratories, Detroit, MI, USA) or Lysogeny broth (LB) media were used for cultivation of fungi and bacteria respectively.

### 2.2. Isolation of Different Morphotypes of B. mojavensis Strain PS17

For this purpose, an overnight culture of *B. mojavensis* PS17 grown on LB medium was inoculated in 2× Schaeffer’s-glucose (2×SG) medium [29,30] and incubated at 36 ± 1 °C under agitation (160 rpm) for 2 weeks. After performing serial dilution to 10^−5^, 0.1 mL aliquots (10^−4^–10^−5^) were plated on 2×SG agar and incubated at 36 ± 1 °C for 5 days. Plates were then visualized under stereomicroscope (Zeiss Jena Technival 2, Gottingen, Germany). The selected colonies with changed morphotypes were reinoculated into a fresh 2×SG medium and then incubated overnight at 35 ± 1 °C. The dilutions of the overnight cultures were plated on LB agar to estimate their phenotypic stability. The presence of colonies with differing morphotypes was considered phenotypic instability in *B. mojavensis* PS17.

### 2.3. Molecular Identification of the Isolates

The identification of the selected strains was performed by PCR based on the comparison of 16S rRNA gene sequences to the National Center for Biotechnology Information (NCBI) GenBank database (accessed on 2 December 2020). The chromosomal DNA was isolated using TRIzol kit (Invitrogen, Carlsbad, CA, USA) according to the manufacturer’s protocol. The 16S rRNA gene was amplified using universal primers 27fm (5’-AGA GTT TGA TCM TGG CTC AG-3’) and R1522 (5’-AAG GAG GTG ATC CAG CCG CA-3’) [31]. The reaction mixture contained 2.5 µL of 10× PCR buffer, 0.4 µL of 10 µM mixture of dNTPs, 1.25 µL of 10 µM each primer, 2.5 µL DNA sample (90 ng), 1.0 μL Taq DNA polymerase (5 U/μL), and free DNA-ase water. PCR conditions were as follows: initial denaturation at 95 °C for 3 min, followed by 36 cycles of denaturation at 95 °C for 15 s, with annealing temperature at 58 °C for 30 s, and elongation at 72 °C for 40 s. The final cycle was followed by an extension at 72 °C for 10 min. The PCR products obtained were fragmented by electrophoresis in 1.5% agarose gel. According to the manufacturer’s recommendations, the purification of DNA fragments from agarose gel was carried out using the Cleanup kit (Evrogen, Moscow, Russia). The nucleotide sequence of the amplified fragments was determined by Evrogen (Moscow, Russia) using both forward and reverse primers. The obtained chromatograms were analyzed, and a consensus sequence was generated using the Clone Manager 9 software package (Sci Ed Software, Cologne, Germany) and blasted on https://blast.ncbi.nlm.nih.gov/Blast.cgi (accessed on 2 December 2020).

### 2.4. DNA Fingerprinting Analysis

DNA fingerprinting was performed according to Versalovic et al. [32] and Louws et al. [33]. For this purpose, the total chromosomal DNA from each bacterial morphotype grown overnight in LB medium at 37 ± 1 °C was isolated as described above in Section 2.3. The primers ERIC1 (5′-GTAAGCTCCTGGGGATT-3′) and ERIC2 (5’-AAGTAAGTGACTGGGGTGAGCG-3′) [32] were used for ERIC-PCR, and the primer BOXAIR (5′-CTACGGCAAGGCGACGCTGACG-3′) for BOX-PCR [33]. The PCR reaction was carried out in a 25 μL volume containing 2.5 µL of 10x PCR buffer, 0.4 µL of 10 µM mixture of dNTPs, 1.25 µL of 10 µM each primer, 5.0 µL DNA template (50 ng), 1.0 μL of Taq DNA polymerase (5 U/μL), and free DNA-ase water. All the reagents used for the master mix were acquired from Evrogen (Moscow, Russia). The amplification was carried out using a T100 thermocycler (Bio-Rad, USA) with the following protocols: initial denaturation at 95 °C for 3 min followed by 35 cycles of denaturation at 95 °C for 1 min; primer-annealing temperature set at 49 °C for 1 min for ERIC-PCR and elongation at 72 °C for 5 min, BOX-PCR with an initial denaturation at 95 °C for 2 min, followed by 30 cycles at 94 °C for 30 s; and primer annealing at 58 °C for 30 sec at 50 °C for 1 min and at 72 °C for 8 min. The final cycle was followed with an extension at 72 °C for 10 min. The PCR product was separated by electrophoresis in 1.5% agarose gel. The agarose gels were observed under ultraviolet light, analyzed, and photographed using Gel Doc EZ Imager with Image Lab 6.0 software (Bio-Rad, Hercules, CA, USA).

### 2.5. Transmission Electron Microscopy

For the ultrastructural examination under transmission electron microscopy (TEM), the bacterial cells were harvested by centrifugation 3000× *g* for 5 min at 4 °C to avoid cell damage. The samples were fixed in 2.5% glutaraldehyde prepared in 0.1 M phosphate buffer (pH 7.2) for 2 h. Then the cells were washed three times with 0.1 M phosphate buffer and post-fixed by incubation in 1% osmium tetroxide for 2 h. After dehydration in an ethanol series (30%, 40%, 50%, 60%, 70%, 80%, 90%, and then 96% ethanol), the specimens were transferred to 100% acetone and propylene oxide. Thereafter, the samples were immersed in Epon resin (Fluka, Buchs, Switzerland) that contained propylene oxide added in proportions of (*v*/*v*) 1:2, 1:1, and 2:1 for 12-h each step. The samples were embedded in pure Epon resin. Ultra-thin sections were prepared using an ultra-microtome Leica UC7 (Wetzlar, Germany), mounted on copper grids, and stained with 2% aqueous uranyl acetate (*w*/*v*) for 20 min and Reynolds’ lead citrate for 7 min. Finally, the sections were observed under a Hitachi HT 7700 Excellence (Tokyo, Japan) at an accelerating voltage of 100 kV.

### 2.6. Bacterial Inoculation and Bacterial Suspension Preparation

The bacterial suspension was prepared from the bacterial culture of *B. mojavensis* morphotypes grown overnight in LB medium at 28 ± 1 °C. Each culture of bacterial morphotype was centrifuged at 4000 rpm for 5 min at 4 °C. The obtained precipitates were washed twice with sterile phosphate-buffered saline (PBS) (140 mM NaCl, 5 mM KH_2_PO_4_, 1 mM NaHCO_3_, pH 7.4) and resuspended in the same solution to an optical density (OD) value of 0.1 at 595 nm.

### 2.7. Growth Quantification

To determine the growth rate, a bacterial suspension of each *B. mojavensis* PS17 morphotype was inoculated in a fresh LB broth medium (g/L) [(tryptone,10 g; yeast extract, 5 g; NaCl, 10 g and minimal medium M9 (g/L) [(5×M9 Salts–(Na_2_HPO_4_7H_2_O, 64 g; KH_2_PO_4_, 15 g; NH_4_Cl, 5 g; NaCl, 2.5 g), 200 mL; 20% glucose, 20 mL; 1M MgSO_4,_ 2 mL; 1M CaCl_2_, 0.1 mL] in a 1:50 ratio. The cultures were incubated in three replicates using 96-well cell culture plates (Costar, New York, NY, USA) for 24 and 40 h at 28 ± 1 °C. The growth rate was determined by measuring the optical density of the culture once per well per hour at 595 nm using a spectrophotometer (SpectrostraNano BMG Labtech, Ortenberg, Germany).

### 2.8. Measurement of Lysis Rates

Lysis rates were measured based on the quantification of free DNA released during the incubation period at 5 °C for one week. For this purpose, each morphotype’s bacterial culture (strain growth in LB medium for 12 h at 28 ± 1 °C) was set to an optical density value of 3 at 595 nm and incubated at 5 °C for 7 days. After 24 h of each incubation period, 1 mL was taken from each flask into 1.5 mL Eppendorf tubes and centrifuged at 14,000 rpm for 5 min at 23 °C. The supernatant (500 µL) was pipetted into new tubes and 300 µL of phenol was added vortexed for 10 sec with the subsequent addition of chloroform (600 µL). The obtained suspension was mixed and centrifuged at 14,000 rpm for 5 min at room temperature. The upper aqueous phase was transferred into a sterile 1.5 mL tube. Isopropanol was added (0.7 volume of aqueous phase), gently mixed, and centrifuged at 14,500 rpm for 5 min. The resulting pellet was washed twice with 70% ethanol, centrifuged at 10,000 rpm, and resuspended in 50 µL nuclease-free water. Nanodrop 2000 c was used to measure the DNA concentration. For the viability of the obtained results, measurement was performed in triplicates.

### 2.9. Activity against Phytopathogenic Fungi

The antagonistic activity was carried out using the method of dual culture on a Potato Dextrose Agar (PDA). The inhibitory effect of *B. mojavensis* PS17(I) and PS17(II) was evaluated using their cell suspension, cell-free supernatant, and lipopeptide extracts. The cell suspension was obtained after centrifugation at 4000 rpm for 10 min of overnight culture at 28 ± 1 °C (Figure S3). The sediments were washed three times with sterile PBS and resuspended in the same solution. The cell-free suspension was obtained after centrifuging at 10,000 rpm for 15 min and aseptic filtration (through a 0.22 µm pore-size membrane filter) of each morphotype of *B. mojavensis* PS17 grown in LB medium at 35 ± 1 °C for 4 days. Lipopeptide extract was obtained after precipitation of cell-free suspension by adding 3N HCl to a final pH of 2 with subsequent overnight storage at 4 °C. The precipitates were then obtained by centrifugation at 10,000 rpm for 20 min at 4 °C followed by dissolution in different solvents (1:1 *v*/*v* diethyl ether, 1:1 *v*/*v* toluene, 1:1 *v*/*v* methanol, and 1:1 *v*/*v* methanol/chloroform (2:1)). The obtained solvents were evaporated, and the resulting precipitates were dissolved in 200 µL of 0.5% DMSO.

The mycelium agar discs of one-week-old fungal colonies (*Fusarium graminearum*, *Fusarium chlamydosporum*, *Fusarium solani*, *Verticillium dahliae*, *Fusarium* spp, and *Fusarium oxysporum* f. sp*. radices-lycopersici* (*Forl*) ZUM2407) were placed at the center of the PDA agar plates. Plates were incubated at 28 ± 1 °C for 2 days. After that, lipopeptide extract, cell suspension, and cell-free supernatant were concentrated in sterile cotton wool discs and plated at 2 cm from fungal mycelium agar discs. Plates were then reincubated at 28 ± 1 °C for 7 days. *Bacillus amyloliquefaciens* REC–95B and *P. putida* PCL1670 were used as positive and negative controls, respectively.

### 2.10. Effect of Phenotypic Variation of B. mojavensis PS17 on Hydrolytic Enzymes and Indole-3-Acetic Acid Production

#### 2.10.1. Indole-3-Acetic Acid (IAA) Production

The IAA produced by *B. mojavensis* PS17(I) and PS17(II) was determined using the colorimetric method according to Gordon and Weber [34]. For this purpose, each morphotype was grown in LB medium amended with 0.1% L-Tryptophan as the precursor of IAA at 28 ± 1 °C with constant shaking (180 rpm) for 3 days. After incubation, bacterial cultures were centrifuged at 10,000 rpm for 10 min at 4 °C. One milliliter of each supernatant was mixed with Salkowski’s reagent (1:4) [35]. The mixture was incubated in a dark place at 28 ± 1 °C for 30 min, and then absorbance was measured at 530 nm. The concentration of IAA produced was estimated using a standard IAA curve (0–1000 μg/mL). One milliliter of sterile LB mixed with Salkowski’s reagent was used as a blank.

#### 2.10.2. Hydrolytic Enzymes Production

##### Bacterial Culture Suspensions

Bacterial culture suspensions were prepared as described above in Section 2.6 with slight modification. In this case, the precipitates were washed 4 times with a sterile PBS and resuspended in the same solution to an optical density value of 0.5 at 595 nm.

For cellulase, protease, and β-glucanase activities, bacterial culture suspensions of each morphotype were inoculated into a minimal medium (g/L: K_2_HPO_4_, 5.8 g; KH_2_PO_4_, 3.0 g; (NH4)_2_SO_4_, 1.0 g; MgSO_4_·7H_2_O, 1.5 g) amended with 1% of carboxymethyl cellulose (CMC), skim milk powder, and β-glucan as carbon sources, and incubated at 37 ± 1 °C for three days with a constant agitation of 160 rpm. After incubation, the bacterial cultures were centrifuged at 10,000 rpm for 12 min at 4 °C. The obtained supernatant was used as a crude enzyme source. A sterile medium was used as blank. Protease activity was carried out using azocasein as substrate, according to Silva et al. [36]. Cellulase activity was performed according to Ghose et al. [37]. The activity of glucanase was determined using the DNS method according to Denault et al. [38] and Ghose et al. [37].

### 2.11. Endophytic Abilities of B. mojavensis PS17 Morphotypes Strain

The endophytic ability of the selected bacterial morphotypes was determined using a gnotobiotic system [39]. Sterile sand pre-moistened with the plant nutrient solution [PNS (g/L): 5.0 mM Ca(NO_3_) 2.4 H_2_O, 1.18 g; 5.0 mM KNO_3_, 0.5 g; 2.0 mM MgSO_4_ 7H_2_O, 0.48 g; pantothenic acid solution, 1.0 mL] was used as a substrate in gnotobiotic systems and was autoclaved. For this purpose, a spontaneous rifampicin mutant of each morphotype was obtained by gradual selection of resistant isolate after cultivation on the lower concentration of rifampicin in LB agar medium with a steady increase in the rifampicin dose. Cell-inoculating suspension was prepared as described above in Section 2.6. Tomato seeds were surface-sterilized according to Simons et al. [39]. Seeds were inoculated for 15 min in cell suspension and then sown in the gnotobiotic system. Plants were grown for 12 days in a climate chamber HPP (Memmert, Germany) with a constant temperature of 25 ± 1 °C, under 90% light, and in 16:8 day: night cycles with 60% humidity. After 12 days, grown seedlings were surface-sterilized, according to Simons et al. [39]. The surface-sterilized roots and shoots were homogenized separately in a sterile mortar and pestle with 1 mL of sterile saline solution (0.9% NaCl). The obtained suspensions (0.1 mL) were plated on LB agar amended with rifampicin (50 µg/mL) and incubated at 28 ± 1 °C for 12 h.

### 2.12. Plant Growth-Promoting Potential of B. mojavensis PS17 Morphotypes Strains

The pot experiment was carried out to evaluate the plant growth-promoting ability of each morphotype strain. The bacterial suspension was prepared from an overnight *B. mojavensis* PS(I), PS17(II), and *P. putida* PCL1760 culture grown in LB medium at 28 ± 1 °C. For this purpose, an equal volume of each culture was inoculated in a ratio of 1:50 into a 20-fold diluted LB medium with sterile tap water. The culture was incubated overnight at 28 ± 1 °C. Bacterial cultures were then centrifuged at 8000 rpm for 15 min at 4 ± 1 °C. The obtained pellet was rinsed twice with sterile PBS and diluted with 2% carboxymethylcellulose (CMC) solution to an optical density value of 0.1 at 595 nm. Non-sterile tomato seeds were inoculated in each cell suspension for 25 min and dried in a laminar flow hood. Seeds were sown into 36.5 cm length × 13.5 cm width × 12 cm depth pots (70 seeds per pot in three repeats per treatment) containing a mixture (1:1) of the garden and bulk soil moistened with distilled water to 60% of its water-holding capacity. Pots were placed in the growth chamber with a temperature of 26 ± 1 °C, under 90% light, and with 16:8 day: night cycles with 70% humidity. For the control group, seeds were inoculated in 2% CMC (negative control) or cell suspension of *P. putida* PLC1760 (positive control). Pots were watered with tap water twice per day (12 h set). After 3 weeks of cultivation, plant biometric parameters such as roots’ length, shoot length, and fresh mass were measured.

### 2.13. Biocontrol Properties of B. mojavensis PS17 Morphotypes on Tomato Plant against Forl ZUM2407

The ability of each morphotype to protect tomato plants against *Forl* ZUM2407 was determined under laboratory conditions in pots containing Rockwool presoaked in a spore suspension of *Forl* ZUM2407 as described in our previous work [26]. Inoculum preparation was prepared as described above in Section 2.12. Seed inoculation was carried out by inoculation for 15 min in bacterial suspensions of PS17(I) and PS17(II). Containers were placed in the dark to facilitate germination and then incubated for up to 28 days under 90% light and 16:8 day:night cycles. For statistical analysis, 90 plants of each group were analyzed. The disease index (DI) was calculated using a scale characterizing the development of the disease severity on the plant according to the Formula [40]:

(1) $$\mathrm{DI}\left(\%\right)=\frac{\left[\left(n_{0}\times 0\right)+\left(n_{1}\times 1\right)+\left(n_{2}\times 2\right)+\left(n_{3}\times 3\right)+\left(n_{4}\times 4\right)\right]}{\left(n_{0}+n_{1}+n_{2}+n_{3}+n_{4}\right)}\times 100$$

where *n*_0_, *n*_1_, *n*_2_, *n*_3_, and *n*_4_ are the number of plants with indices 0, 1, 2, 3, and 4, respectively.

### 2.14. Statistical Analysis

Statistical data analysis was performed using the statistical program originLab pro SR1 b9.5.1.195 (OriginLab Corp., Northampton, MA, USA). The significant difference between groups was analyzed using one-way ANOVA and post hoc Tukey’s honestly significant difference test (*p* < 0.05).

## 3. Results

### 3.1. Morphology of Phase Variability and Verification of the Phasal Leniage

**Figure 1:**
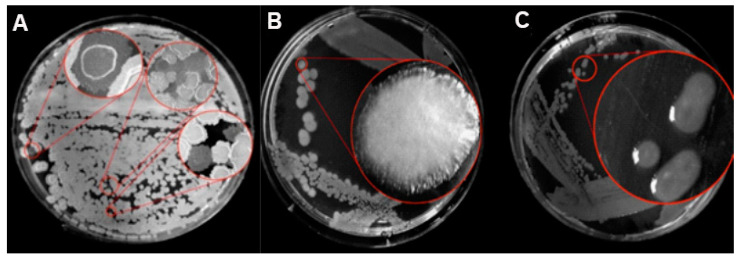
The occurrence of phase variation in *B. mojavensis* PS17 (**A**). *B. mojavensis* PS17 morphotype I (**B**) and morphotype II (**C**) grown on 2×SG agar medium plate. Red circles show magnified colonies from specific sites on plates.

**Figure 2:**
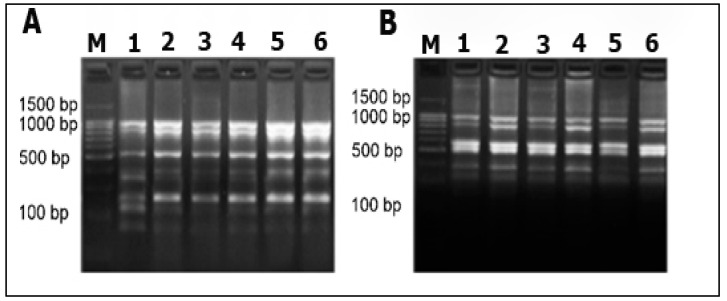
The 1.5% agarose gel electrophoresis of representative (**A**) BOX and (**B**) ERIC PCR patterns. M—DNA ladder; 1–2—*B. mojavensis* PS17(I); 3–4—*B. mojavensis* PS17(II); 5–6—the original strain of *B. mojavensis* PS17.

It was observed that *B.* *mojavensis* PS17 grown in 2×SG produced translucent colonies (Figure 1, Figures S1 and S2). These translucent colonies of strain PS17 are presented in Figure 1C. The results revealed that both isolates generated identical BOX and ERIC fingerprint profiles in comparison to the original strain (Figure 2). Based on the similarity of the generated BOX and ERIC DNA fragments, both isolated morphotype strains genetically belong to *B. mojavensis* PS17. The original strain of *B. mojavensis* PS17 (NCBI accession number MW350040) with an opaque morphology of its colonies was designated as strain PS17 morphotype I or in brief PS17(I), whereas translucent type colonies were designated as strain PS17 morphotype II or in brief PS17(II). These translucent colonies of strain PS17 (Figure 1C) appear in each plated single colony from normal opaque morphology and were 99% identical to *B. mojavensis* based on 16S rRNA gene sequence analysis. The sequence of the 16S rRNA gene fragment of the translucent colony was deposited in the NCBI GenBank database with the accession number MW350045 as *B. mojavensis* PS17 morphotype II. To confirm both morphotypes as the original PS17 strain, BOX and ERIC PCR fingerprinting were performed.

### 3.2. Structural Analysis under Transmission Electron Microscopy (TEM)

**Figure 3:**
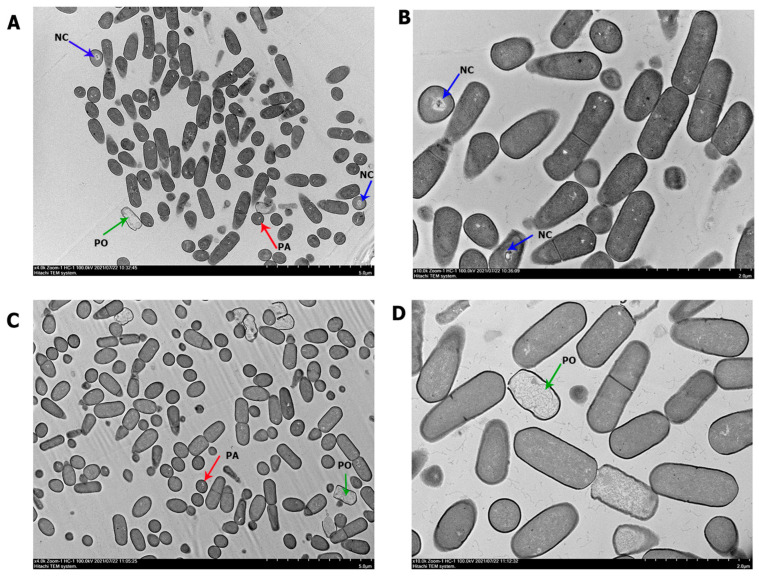
Transmission electron microscopy images of *B. mojavensis* PS17(I) (**A**,**B**) and *B. mojavensis* PS17(II) (**C**,**D**) under phenotypic variation. Red arrows—cell with electron-transparent inclusion cytoplasmic membrane (PA); Blue arrows—condensation of cell nucleoid (NC); green arrows—plasmolyzed cells (PO). Image scale: 5 µm (**A**,**C**); 2 µm (**B**,**D**).

To reveal differences at the cellular level, TEM was used to evaluate the cell morphology of *B. mojavensis* PS17(I) and PS17(II). A significant difference in morphological structure was observed (Figure 3). Cells of both morphotypes were heterogenous and demonstrated a typical size range from 1.35 ± 0.158 to 1.64 ± 0.26 µm in length and 0.61 ± 0.051 to 0.73 ± 0.07 µm in diameter (Table 2). A more developed cytoplasmic density was observed in cell PS17(I) (Figure 3A,B). The cytoplasmic matrices of the cells of *B. mojavensis* PS17(II) (Figure 3C,D) are almost electron-dense as compared to the cells of *B. mojavensis* PS17(I) showing electron-translucent cells of PS17(II). The population of cells was heterogeneous in both morphotypes. The first group of cells was characterized by an electron-dense fine granular cytoplasm with evenly distributed nucleoids. Electron-transparent inclusions such as polyalkanoates (red arrows) were seen in the cells with intact outer and cytoplasmic membranes. A group of PS17(I) cells differed from PS17(II) by their enlightened evenly granular structure with electron-dense zones resulting in the condensation of the nucleoid (blue arrows) (Figure 3A,B). There are also groups of cells with enlarged periplasmic spaces with some cells plasmolyzed (green arrows), thereby releasing the remnants of their cytoplasm (Figure 3C,D). The ratio of the cell groups in both morphotypes is represented in Table 2.

### 3.3. Characteristics of Colony Phase Change

#### 3.3.1. Quantitative Assessment of Growth for *B. mojavensis* PS17 Morphotype I and II

**Figure 4:**
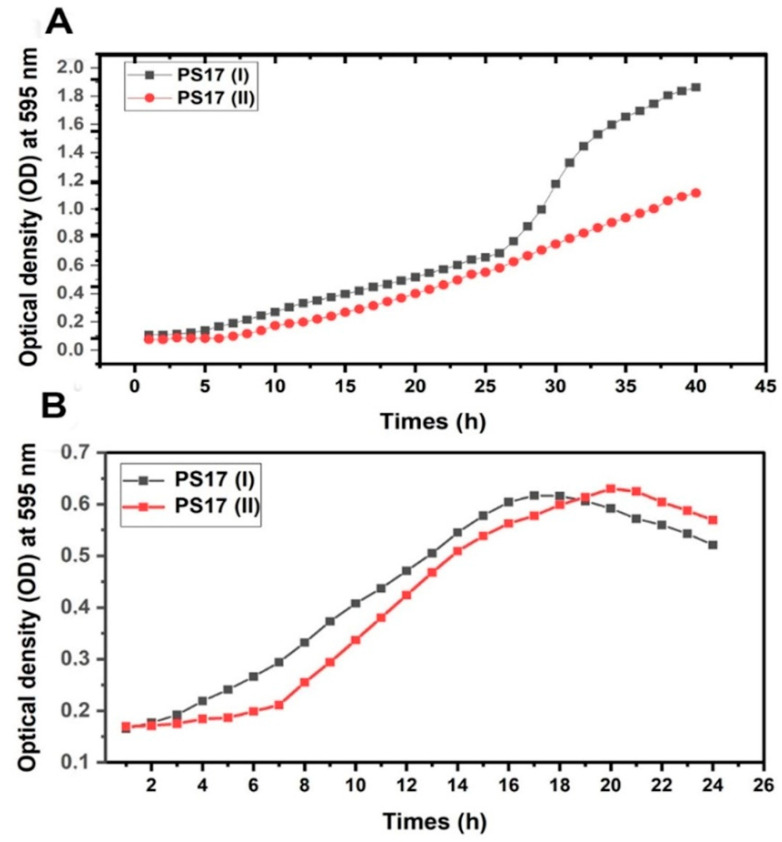
Graph showing growth curve of *B. mojavensis* PS17 morphotypes I and II in M9 medium (**A**) and LB medium (**B**).

The growth curves of *B. mojavensis* PS17(I) and PS17(II) are given in Figure 4A (in a minimal medium, M9) and Figure 4B (in a rich nutrient medium, LB). When inoculated in a minimal M9 medium, the growth rates of *B. mojavensis* PS17(I) and *B. mojavensis* PS17(II) were almost identical in the lagging and exponential phases from the 5th h to the 26th h with an average OD difference of 0.1 (Figure 4A). The difference in the growth rates was noticeable only after 26 h of incubation, where the culture of PS17(I) grew more intensively than the culture of PS17(II) (Figure 4A). The maximum optical density in the logarithmic phase of *B. mojavensis* PS17(II) was 0.749 less than the value of *B. mojavensis* PS17(I). In the rich LB medium (Figure 4B), *B. mojavensis* PS17(II) registered an exponential growth phase immediately after the first hour of incubation that lasted up to 16 h. *B. mojavensis* PS17(II) recorded an exponential growth phase only after 6 h of incubation (lag phase), reaching maximum growth 2 h into the death phase of *B. mojavensis* PS17(I).

#### 3.3.2. Measurements of the Autolytic Rate of Morphotypes

**Figure 5:**
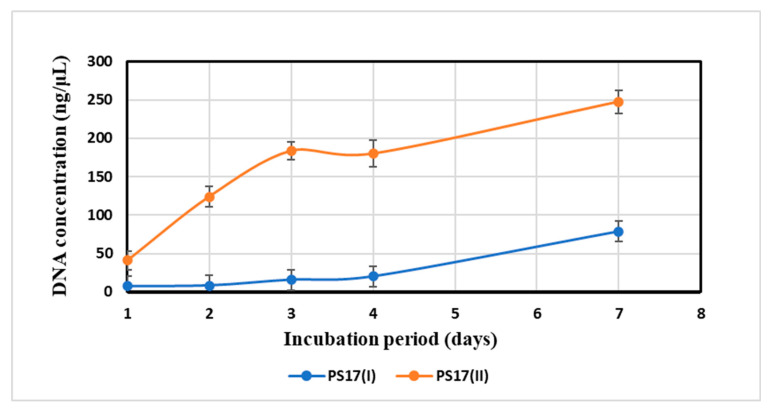
Cell lysis rate of *B. mojavensis* PS17 morphotypes.

As observed from the results given in Figure 5, the autolytic activity of morphotypes I and II statistically differed (*p* < 0.05). Their rates increased proportionally with the incubation times (Figure 5). *B. mojavensis* PS17(I) released free DNA concentrations ranging from 7.7 to 78.5 ng/μL, whereas *B. mojavensis* PS17(II) released up to 247.5 ng/μL. The obtained results indicated that *B. mojavensis* PS17(II) has a higher autolytic rate than *B. mojavensis* PS17(I).

#### 3.3.3. Activity against Phytopathogenic Fungi

**Figure 6:**
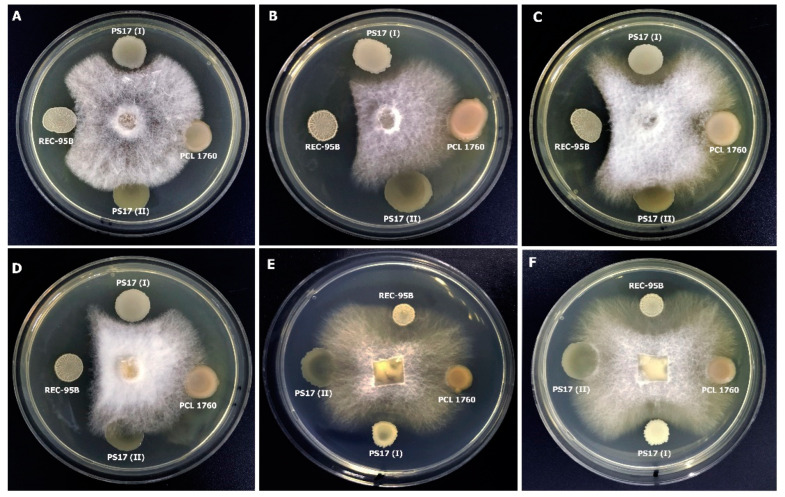
Antifungal activity of the bacterial cell suspension against phytopathogenic *V. dahliae* (**A**)*, F. chlamydosporum* (**B**), *Fusarium spp.* (**C**)*, F. solani* (**D**), *F. graminearum* (**E**), and *Forl* ZUM2407 (**F**).

**Figure 7:**
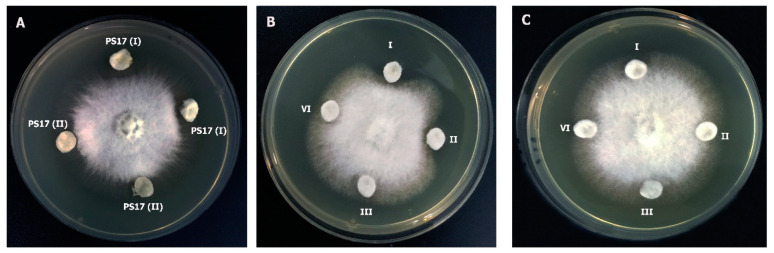
Antifungal activity of cell-free suspension (**A**) obtained from *B. mojavensis* PS17(I) and *B. mojavensis* PS17(II). Antifungal activity of lipopeptides extracted from (**B**) *B. mojavensis* PS17(I) and (**C**) *B. mojavensis* PS17(II) against phytopathogenic fungi *Forl* ZUM2407. Lipopeptide extracted with Diethyl ether (I), methanol/chloroform (II), toluene (III), and methanol (IV). Cotton wool discs amended with 50 μL of lipopeptide extracts diluted in (0.5%) DMSO were used for antifungal ability.

The results revealed that *B. mojavensis* PS17(II) was unable to inhibit the growth of all the fungal phytopathogens tested in the experiment compared to *B. mojavensis* PS17(I) (Figure 6), which showed a wide range of inhibition activity. Similar results were obtained when the cell-free suspensions and lipopeptides extracted from *B. mojavensis* PS17(I) and *B. mojavensis* PS17(II) were tested against *Forl* ZUM2407 (Figure 7). Since the lipopeptide extracts from PS(II) did not show antagonism against the phytopathogens, the antagonism of PS17(I) was due to its secondary metabolites in the cell-free suspension, not DMSO.

### 3.4. Effect of Phenotypic Variation of B. mojavensis PS17 on Hydrolytic Enzymes and Indole-3-Acetic Acid (IAA) Production

To determine whether phase variation had induced a change in the biosynthesis of the secondary metabolites, the abilities of *B. mojavensis* PS17(I) and *B. mojavensis* PS17(II) to produce indole-3-acetic acid and hydrolytic enzymes were evaluated.

#### 3.4.1. Indole-3-Acetic Acid (IAA) Production

**Figure 8:**
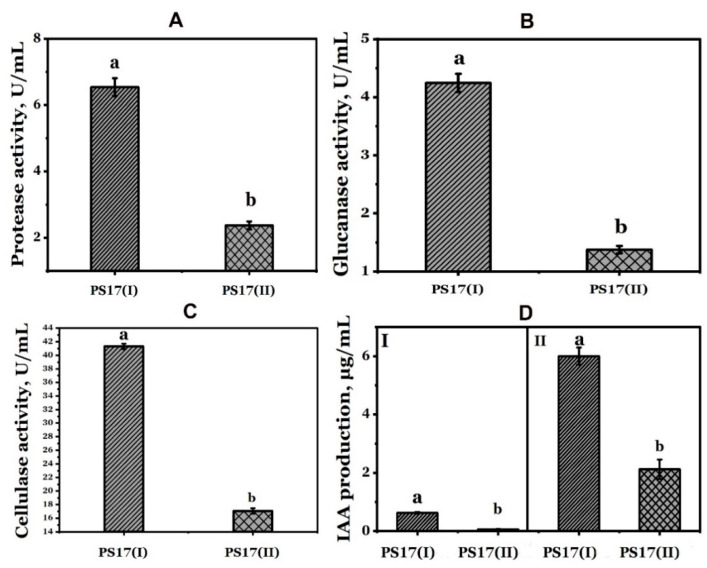
Effect of phenotypic variation on hydrolytic enzyme activity ((**A**) protease, (**B**) glucanase, (**C**) cellulase), and (**D**) Indole-3-acetic acid production (I-without tryptophan; II-with tryptophan) of *B. mojavensis* PS17. The experiment was performed in triplicates and repeated twice. Statistical differences at *p*-value < 0.05 between groups are indicated by different letters.

The obtained results are presented in Figure 8D. As it can be observed, both morphotypes showed auxin production potential in the presence of tryptophan. After incubation, auxin production by *B. mojavensis* PS17(I) was measured at 6.01 μg/mL, while the value of *B. mojavensis* PS17 (II) was measured at 2.12 μg/mL. The IAA produced by *B. mojavensis* PS17 (II) was 64.72% less than PS17(I) (Figure 8D, II). In the absence of a tryptophan precursor, IAA was not produced by *B. mojavensis* PS17(II), in contrast with PS17(I), in which the value was measured as 0.67 μg/mL (Figure 8D, I).

#### 3.4.2. Hydrolytic Enzymes Production

The hydrolytic enzyme production such as protease, cellulase, and β-glucanase was also evaluated. The obtained results revealed a decrease in the production of these enzymes from *B. mojavensis* PS17(II) compared to strain PS17(I). The enzyme activity of the proteases produced by *B. mojavensis* PS17(II) was 3.22 times less than that of *B. mojavensis* PS17(I) (Figure 8A). Their activities were assessed as 6.44 ± 0.25 U/mL and 2.26 ± 0.11 U/mL. Similar results were obtained with the cellulase and β-glucanase activities’ assessments. Cellulase and β-glucanase produced by *B. mojavensis* PS17(I) were up to 2.42 and 3.09-fold more active, respectively, than *B. mojavensis* PS17(II). The cellulase and β-glucanase activities of *B. mojavensis* PS17(I) were measured as 41.31 ± 0.39 U/mL and 4.24 ± 0.16 U/mL, respectively, and those of *B. mojavensis* PS17(II) as 17.06 ± 0.41 U/mL and 1.37 ± 0.06 U/mL.

### 3.5. The Endophytic Ability of B. mojavensis PS17 Morphotypes

**Figure 9:**
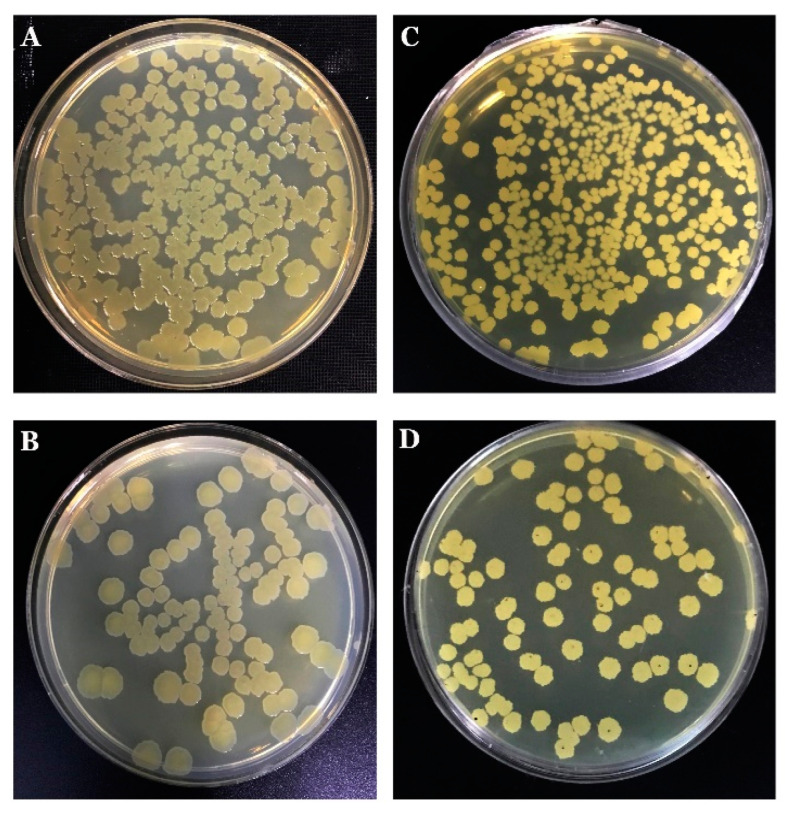
The endophytic ability of *B. mojavensis* P17 morphotypes I and II to colonize tomato plants. *B. mojavensis* P17(II) isolated from root (**A**) and shoot (**B**) surface-sterilized growth on LB medium. *B. mojavensis* PS17(I) isolated from root (**C**) and shoot (**D**) surface-sterilized growth on LB medium.

Based on their morphological characteristics and ability to grow on a selective medium (Figure 9), it was distinguished that the grown bacteria belonged to the inoculated original strains (*B. mojavensis* PS17(II) and PS17(I)). In addition to this, it can be observed in Figure 9 that both morphotypes did not lose their endophytic ability. Even during colonization, the reverse switches of B. mojavensis PS17 (II) to *B. mojavensis* PS17 (I) were not observed.

### 3.6. Plant Growth-Promoting Potential of B. mojavensis PS17 Morphotypes

**Figure 10:**
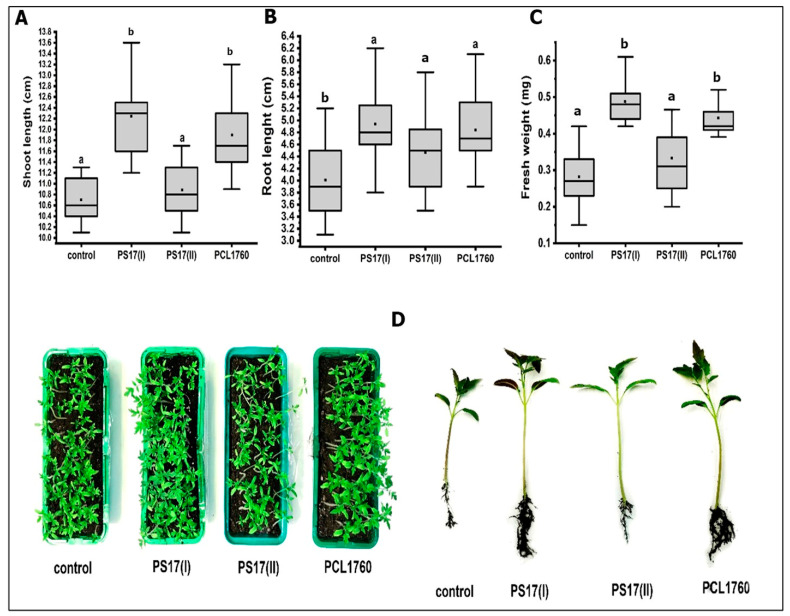
Plant growth-promoting activity of *B. mojavensis* PS17 morphotypes on (**A**) shoots, (**B**) roots, and (**C**) fresh weight of tomato plants. (**D**) Photo recording tomato plant growth 3 weeks after seed sowing. The experiment was performed in triplicate and repeated twice. Statistical differences at *p*-value < 0.05 between groups are indicated by different letters.

The plant growth-promoting effects of each morphotype strain on tomato plants were evaluated by the increase in the plants’ biometric parameters after treatment. Both morphotypes had a positive effect on the tomato plants in comparison to the negative control without treatment (Figure 10). However, there were significant differences in the plant growth-stimulating effects between *B. mojavensis* PS17(I) and the positive control, PCL1670 (Figure 10). The growth parameters (shoot length, root length, and fresh weight) of the plants treated with strain PS17(I) were measured as 12.26 cm, 5.10 cm, and 0.491 g, respectively. Whereas the respective values of the plants treated with strain PS17(II) were 10.89 cm, 4.47 cm, and 0.334 g, compared to the control groups whose plant growth parameters were 10.2 cm, 4.20 cm, and 0.035 g. The growth parameters of the plants treated with strain PCL1760 were 11.91 cm, 4.80 cm, and 0.448 g.

In contrast, no significant difference (*p* < 0.05) was observed between the control (untreated plants) and the plants treated with the strain PS17(II) regarding the shoot length and fresh weight of the plants (Figure 10A,C). Likewise, no significant difference was also observed among the strains PCL1670, PS17(I), and PS17(II) regarding their root length (Figure 10B).

### 3.7. Biocontrol Properties of B. mojavensis PS17 Morphotypes Strains on Tomato Plant against Forl ZUM2407

**Figure 11:**
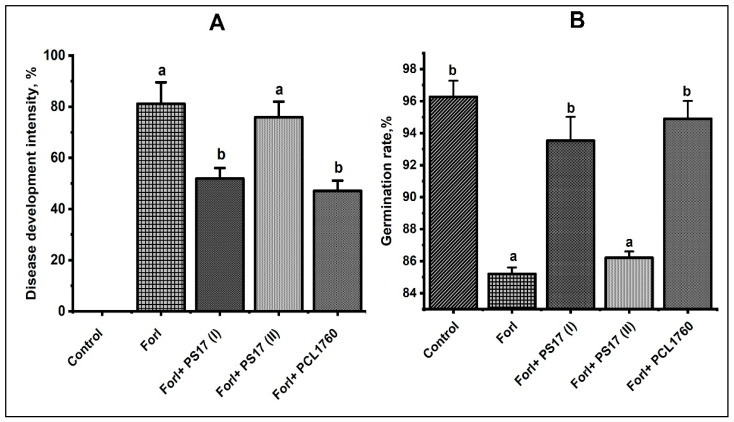
Biocontrol properties (**A**) and germination rates (**B**) of *B. mojavensis* PS17 morphotypes I and II on tomato plants against *ForI* ZUM2407, PS17(I), PS(II), and PCL1670. The experiment was performed in duplicate and repeated twice. Statistical differences between groups are indicated by different letters (a and b) to the least significant difference at a *p*-value < 0.05.

Based on the obtained results of the antagonistic activity in vitro, we tested the potential of *B. mojavensis* PS17(I) and PS17(II) to inhibit root rot of tomato plants caused by *Forl* ZUM2407 in pot experiments. The treatment of tomato seeds with *B. mojavensis* PS17(I) after 28 days demonstrated a significant capacity for biocontrol (Figure 11A) as compared with the negative control group (seed without treatment). The disease progression was statistically different (*p* < 0.05) since the disease index recorded in the group pretreated with *B. mojavensis* PS17(I) was 51.89 ± 1.07% and 81.15 ± 2.16% for the negative control group. There was no statistically significant difference (*p* < 0.05) in the disease progression between the groups inoculated with *B. mojavensis* PS17(I) and *P. putida* PCL1760, which reduced disease development by 47.9 ± 1.03%.

In contrast to the result obtained with *B. mojavensis* PS17(I), no statistically significant difference was found in the suppression of tomato foot and root rot (TFRR) disease caused by *Forl* ZUM2407 between the groups pretreated with *B. mojavensis* PS17(II) and the negative control (*Forl*, Figure 11A) *p*-value < 0.05. The disease progression in the negative control group amounted to 81.15 ± 2.16% whilst the experimental group pretreated with *B. mojavensis* PS17(II) had a 75.8 ± 1.58% with the seeds showing suppression of TFRR.

Identical results were also observed for the germination rate of seeds. The inoculation of the tomato seeds with *B. mojavensis* PS17(I) showed a statistically significant increment in germination in the group pretreated with *B. mojavensis* PS17(I) amounting to 93.53 ± 1.48% in comparison to the negative control group (85.2 ± 0.4%). On the other hand, the inoculation of the tomato seeds with *B. mojavensis* PS17(II) did not statically (*p* < 0.05) increase the germination of seeds as compared to the negative control. The difference in the germination rates between *B. mojavensis* PS17(II) and the negative control was less than 5% (Figure 11B). No statistically significant difference (*p* < 0.05) was found between the healthy control seedlings (Control), the plants pretreated with *B. mojavensis* PS17(I) (*Forl* ZUM2407 + PS17(I)), and *P. putida* PCL1670 (*Forl* ZUM2407 + PCL1670), in which the germination rate was 94.9 ± 1.11% (Figure 11B).

## 4. Discussion

The haploid genome of a bacterial cell as a single functional chromosome has most of its genetic changes induced by environmental stress, with direct effects on their phenotypic properties such as bacterial growth and its inactivity [41]. This suggests that the effectiveness of using a microbial biocontrol primarily depends on their phenotypic stability. Phenotypic variation may induce some changes in microbial ability to generate the desired properties during their long-term existence in an unfavorable condition.

Phase variation or random reversible phenotypic variation can be considered as an adaptive process that mutates bacteria and thereby enables them to thrive under stressful environmental conditions. In our study, two morphotypes were observed when *B. mojavensis* PS17 was grown on a 2×SG spore-forming medium with glucose as the source of carbon, designating the original *B. mojavensis* PS17 as morphotype I and a morphotype with translucent colonies as morphotype II (Figure 1, Figures S1 and S2). A differing result on a rich medium (PAF medium) was presented by Chabeaud et al. [42], where a prolonged (seven-days) growth of *Pseudomonas brassicacerum* NFM421 brought about variants on the edges of colonies. The occurrence of these variants may be due to the simultaneous expression of genes encoding the structure of the bacterial surface (adhesins, pili, lipooligosaccharide, and others) as described in *Haemophilus influenzae* type b strains [43,44,45,46] and capsule phase variation in *Vibrio parahaemolyticus* [47,48].

The reverse switching of PS17(II) to PS17(I) was not observed when PS17(II) was cultivated on the rich LB medium, confirming the permanent mutation in the translucent strain *B. mojavensis* PS17(II). This may be due to phase variation by DNA modifications, such as the recombination mechanism through gene deletion, making them irreversible phenotypes, as described in the pilin gene variation of *Neisseria gonorrhoeae* [49,50]. A similar result was reported by Li et al. [13] whereby *Acidovorax radicis* N35, a plant growth-promoting potential bacteria isolated from surface-sterilized wheat roots, demonstrated irreversible phenotypic variation in nutrient broth due to a 16-nucleotide deletion in a gene encoding the mismatch repair protein *MutL*.

Molecular identification using 16S rRNA of PS17(I) and PS17(II) confirmed their identity to the original *B. mojavensis* PS17 on the NCBI database. Since phase variation is associated with reversible genomic alterations [6], the appropriate approach was to compare these morphotypic variants with the original strain using a sensitive method to identify intraspecies strains such as BOX, ERIC, REP, and RAPD PCR [32]. In the study conducted by Pogorelova et al. [51], BOX and ERIC-PCR fingerprinting were used to observe genomic alterations in *Azospirillum brasilense* (strains Sp7 and SP245), where ERIC-PCR showed identical profiles of the phase variants. In our case, the BOX and ERIC PCR profiles of both morphotypes were identical to the original strain (Figure 3). We suggest that, depending on the genetic mechanisms leading to phase variation [52,53,54,55], changes in the genome did not occur within the sequences that the BOX and ERIC primers target.

The growth curves of these morphotypes showed differences in the exponential phase and stationary phase, where genes are regulated in nutrient-limited environments. A linear exponential phase (Figure 4A) in comparison to a steep exponential phase (Figure 4B) was observed in morphotype II when it was grown in M9, which might be due to a mutation of the *recA* gene as described by Sciochetti et al. [56] in *B. subtilis.* This phenomenon was not observed when both morphotypes were grown in the LB-rich medium. In the LB medium, all four growth phases were observed within 25 h of incubation. This may be due to the ‘ON’ switch of all the housekeeping genes exhausting both phases to acquire a short stationary phase with a quick decline to the death phase as a result of inadequate nutrient intake.

The presence of cells with enlarged periplasmic space and plasmolyzed cells mostly in morphotype II as opposed to morphotype I attest to the quick autolyzing rate of morphotype II, as evident from the high DNA concentration in the supernatant of the 7-day culture after incubation at 4 °C. This may be due to the induced expression of autolysin genes (*lytC*, *lytD*, *lytE*, and *lytF*) of PS17(II), thereby signaling that autolysis as has been reported in *B. subtillis* by Lamsa et al. [57]. In our case, we suggest a phase-variable restriction activity in morphotype II inducing autolysis, thereby releasing DNA into the surrounding environment for uptake by other cells [58]. We also suggest that the mutation in morphotype II triggers the cells to repair through DNA recombination by taking up DNA from lysed cells as reported in the *Streptococcus pneumoniae* strain Rx [59]. Another study on different strains of *Streptococcus pneumoniae* reported a phase-variable *hsdS* allele recombination that resulted in the appearance of opaque and transparent colonies [60]. The transparent colonies underwent faster autolysis by the production of teichoic acid, which might be the reason for the faster autolysis seen in our translucent phase variant (morphotype II) of *B. mojavensis* PS17 [61].

The biosynthesis of secondary metabolites and hydrolytic enzymes by microbial biological control agents are the main factors in plant–microbe interactions, which play a vital role in regulating plant growth and protection in response to abiotic and biotic stress [62]. A low production of hydrolytic enzymes can have a direct effect on the autolysis of bacterial cells. Kodama et al. [63] reported that the mutation in *spo0A* of *B. subtillis* 168 caused the low expression of extracellular enzymes, which brought about an extensive autolysis of the cells. We suggest that a similar mutation took place in morphotype II of *B. mojavensis* PS17, as it shows a low hydrolytic activity of PS17(II) and an extensive autolysis of its cells. Indole-3-acetic acid (IAA) produced by bacteria, for example, stimulates root growth under various conditions and nutrient uptake from the soil [63]. Hydrolytic enzymes secreted by bacteria are directly involved in the lysis of phytopathogenic fungi [64]. The abilities of *B. mojavensis* strains to secrete plant growth modulators are well documented [21,24,26]. Since phenotypic variation leads to reversible genomic alteration, it was reasonable to investigate whether it induces changes in the expression (up-expression or low-expression) of secondary metabolites and hydrolytic enzymes.

A low rate of IAA biosynthesis and the low hydrolytic enzymes secretion by *B. mojavensis* PS17(II) was observed compared to *B. mojavensis* PS17(I) (Figure 8). This phenomenon might have been due to the down-expression of genes involved in IAA and hydrolytic enzyme biosynthesis. *B. mojavensis* PS17(I) showed the biocontrol characteristics of the original strain *B. mojavensis* PS17 [26]. In this case, the mechanisms for phase variation might have inactivated or caused a genetic deletion in the gene clusters or genes responsible for the expression of these metabolites. For example, the loss of the capacity for the biocontrol of *P. fluorescens* strains in the suppression of soilborne plant pathogens has been attributed to deleterious mutations in *lemA*-*gacA*-type genes regulating secondary metabolite biosynthesis [65,66,67]. A similar result was also reported by van den Broek et al. [68], where *Pseudomonas* sp. PCL1171 generated strains lacking antagonistic activity due to a mutation in the gene encoding lipopeptide synthetase. A similar result was also previously reported by Chabeaud et al. [42]. In their study, a phase II variant of *Pseudomonas brassicacerum* NFM421 was not found to produce extracellular protease and lipase due to the non-transcription of *Apra*-*lipA* operon.

An *in planta* analysis is an optimal test in which different modes of action of microbial biological agents can be detected, such as the competition for nutrients and various ecological niches, induced systemic resistance (ISR) or systemic acquired resistance (SAR), and others [69,70]. Bacon and Hilton [24] isolated *B. mojavensis* strains that exhibited different abilities in planta against *F. verticillioides* compared to in vitro. Another example is the study conducted by Besset-Manzoni et al. [71], in which strains without an in vitro inhibition of phytopathogens were more effective in planta. In our present study, the *in planta* assay of *B. mojavensis* PS17(I) and *B. mojavensis* PS17(II) demonstrated different biocontrol abilities (Figure 11). *B. mojavensis* PS17(II) showed a poor capacity for biocontrol (less than 5%) compared to *B. mojavensis* PS17(I), in which the capacity for biocontrol was up to 50% effective with respect to the suppression of tomato foot and root rot caused by *Forl* ZUM2407 (Figure 11A). As a supporting result, Troxler et al. [72] found that the biocontrol *P. fluorescens* strain CHAO isolated from the root of maize (*Zea mays* L.) exhibited different phenotypic characteristics than those isolated from the rhizosphere. Barnett et al. [73] found that the inhibition of fungi by the microbial biocontrol agent *Pseudomonas corrugate* 2140 was altered by phenotypic variants isolated from wheat roots.

In contrast to *B. mojavensis* PS17(II), *B. mojavensis* PS17(I) showed a similar biocontrol and plant growth ability to strain PCL1760. The proprieties of PCL1760 that demonstrate its capacity as a good biocontrol agent include its ability to synthesize plant growth-regulating metabolites such as cytokinins, auxins, and gibberellins to promote nutrient uptake from the soil through different mechanisms—such as phosphorus solubilization, nitrogen fixation, and siderophores—and for its nutrient niche competitiveness [74,75]. In this context, the biosynthesis of secondary metabolites can be presented as the main mode of action used by *B. mojavensis* PS17 to control diseases caused by phytopathogens such as *F.* *graminearum*, *A. alternate*, *F. chlamydosporum*, *E. nigrum*, and *Forl* ZUM2407. We plan to investigate and confirm the genetic changes associated with the phenotypic variation of *B. mojavensis* PS17 through full genome sequences and comparative analysis of the two phase variants.

## 5. Conclusions

Phase variation is a phenomenon that is associated with the occurrence of different phenotypes of a strain in response to changing environmental conditions. This phenomenon does take place in natural habitats and can be studied under laboratory conditions. In this work, we observed that *B. mojavensis* PS17, a close relative species of *B. subtilis*, undergoes phase variation forming two colonies: opaque (morphotype I) and translucent (morphotype II). The phenotypic characteristics of morphotype II showed the isolate to be less stable in comparison to morphotype I, which could be a cause of the low efficiency of biocontrol agents in crop protection. This phenomenon should be considered in the laboratory before mass production and application. The dynamic mechanisms of phase variation induce changes in the original properties of BCA required for the biocontrol of phytopathogens and plant growth promotion. On the other hand, phenotypic variation can be considered an opportunity to optimize strains for a particular niche, plant type, and soil condition. An in-depth study of this phenomenon can help to understand ways to prevent this switch and increase the effectiveness of bio-inoculants. A genomic comparison of a wild strain with phenotypic variants and their stability in field conditions will help underline all the types of molecular and genetic changes induced by this phenomenon.

## Figures and Tables

**Table 1:** Strains used in this study.

Strain Name	Description	Source/Reference
*B. mojavensis* PS17, referred to as morphotype I	Biocontrol agent uses the mechanism of antibiosis for plant protection	Diabankana et al. [26] Collection of Agroecological Research center of Kazan State Agrarian University Deposited in All Russian Collection of Industrial Microorganisms as *Bacillus mojavensis* B-13415
*B. mojavensis* PS17 morphotype II, referred to as morphotype II	Morphotypic variant of *B. mojavensis* PS17 forming translucent colonies on 2× Schaeffer’s agar surface.	Isolated in this work
*Pseudomonas putida* PCL1760	The biocontrol agent uses the mechanism of completion for nutrients and niches (CNN). In this work, it is used as a reference biocontrol strain in biocontrol assay of tomato plantlets.	Validov et al. [27] Deposited in All Russian Collection of industrial microorganisms as *Pseudomonas plecoglossicida* B-13802
*Fusarium oxysporum* f.sp. *radices-lycopersici* (*Forl*) ZUM2407	The causal agent of foot and root rot of tomato.	Validov et al. [28]

**Table 2:** Characterization of *B. mojavensis* PS17 cells observed under TEM after phase variation.

Cell Characterization		PS17 (I)	PS17 (II)
Length (µm)	Minimum	1.09	1.17
	Maximum	1.54	2.09
	Average ± stdev	1.35 ± 0.158	1.64 ± 0.26
Diameter (µm)	Minimum	0.512	0.624
	Maximum	0.676	0.874
	Average ± stdev	0.61 ± 0.051	0.73 ± 0.07 ^a^
Plasmolyzed cells		53 (15.18%) of 349 cells counted	54 (19.35%) of 279 cells counted
Cells with enlarged periplasmic		28 (8.02%) of 349 cells counted	32 (11.46%) of 279 cells counted

N.B. stdev—standard deviation; (^a^)—significant difference between PS17(I) and PS17(II) at *p*-value < 0.05.

## Data Availability

Not applicable.

## Supplementary Materials

The following supporting information can be downloaded at: https://www.mdpi.com/article/10.3390/biology11091305/s1. Figure S1: Phenotypic variation in *B. mojavensis* PS17. Red arrows indicated translucent colonies of *B. mojavensis* PS17. Figure S2: B. *mojavensis* PS17 observed under a stereomicroscope (Zeiss Jena Technival 2, Germany). Red arrows indicated translucent colonies of *B. mojavensis* PS17, Figure S3: *B. mojavensis* PS17(I) (A) and PS17(II) (B) grown in LB medium.
