# Supplementary material for: Effects of Phenotypic Variation on Biological Properties of Endophytic Bacteria Bacillus mojavensis PS17
Source: Biology (Basel). 2022 Sep 2;11(9):1305. doi: 10.3390/biology11091305 (PMC9495571; doi:10.3390/biology11091305)
Supplement: Supplementary file 1 [file biology-11-01305-s001.zip › biology-1876606-supplementary.pdf]

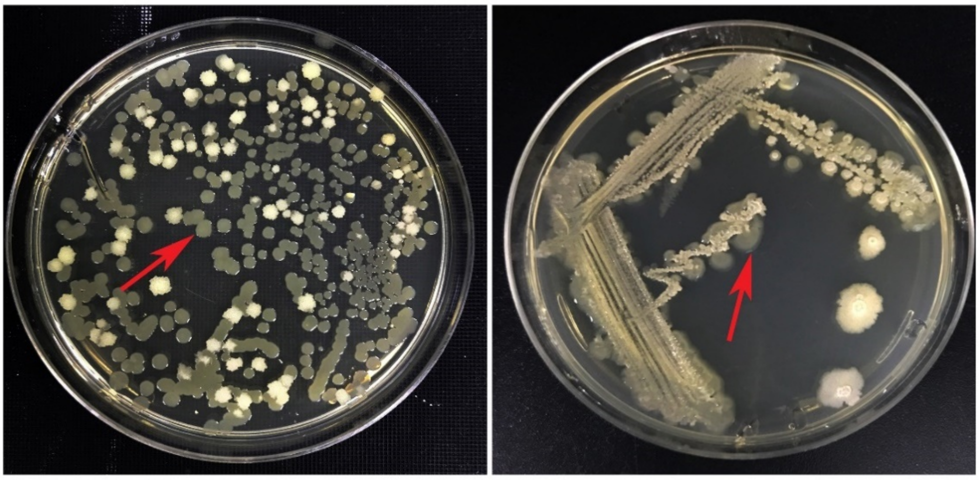

Figure S1. Phenotypic variation in *B. mojavensis* PS17. Red arrows indicated translucent colonies of *B. mojavensis* PS17.

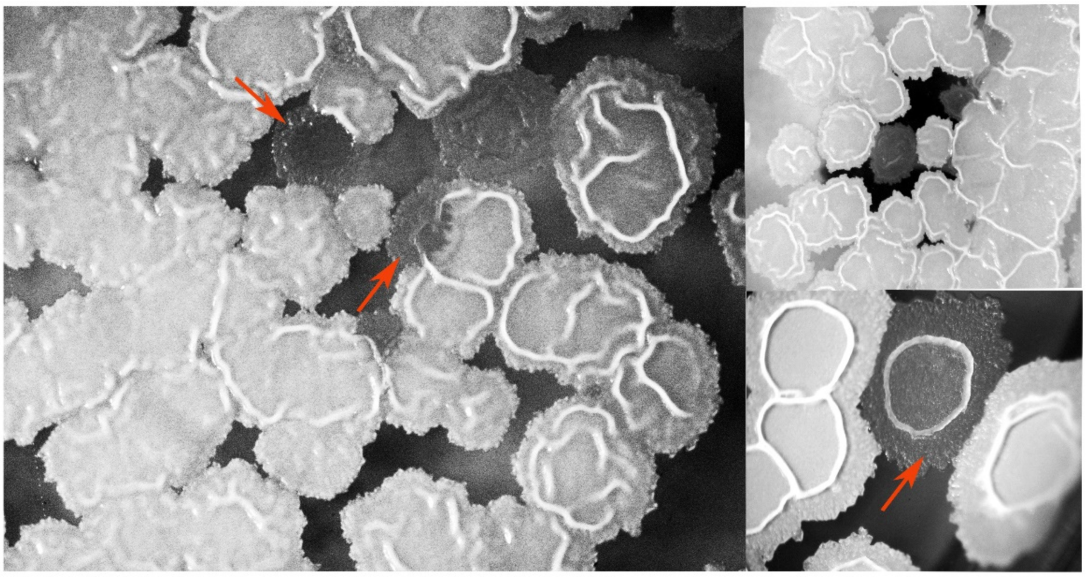

Figure S2 B. *mojavensis* PS17 observed under stereomicroscope (Zeiss Jena Technival 2, Germany). Red arrows indicated translucent colonies of *B. mojavensis* PS17.

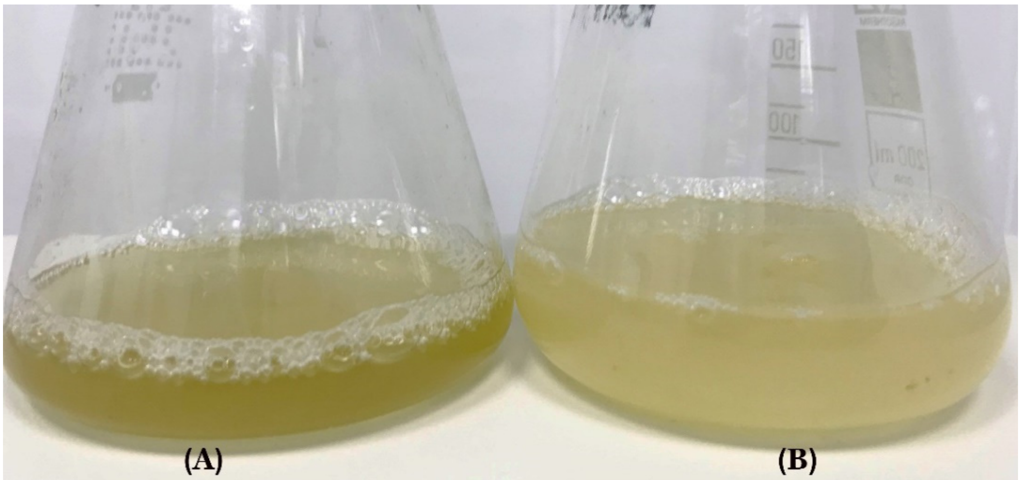

Figure S3 *B. mojavensis* strain PS17(II) (A) and PS17(I) (B) grown in LB medium
